# Real World Experience of Crizotinib in 104 Patients With ALK Rearrangement Non-small-cell Lung Cancer in a Single Chinese Cancer Center

**DOI:** 10.3389/fonc.2019.01116

**Published:** 2019-10-22

**Authors:** Chang Liu, Hui Yu, Qianqian Long, Haiquan Chen, Yuan Li, Weixin Zhao, Kuaile Zhao, Zhengfei Zhu, Si Sun, Min Fan, Jianhua Chang, Jialei Wang

**Affiliations:** ^1^Department of Medical Oncology, Fudan University Shanghai Cancer Center, Shanghai, China; ^2^Department of Oncology, Shanghai Medical College, Fudan University, Shanghai, China; ^3^Department of Thoracic Surgery, Fudan University Shanghai Cancer Center, Shanghai, China; ^4^Department of Pathology, Fudan University Shanghai Cancer Center, Shanghai, China; ^5^Department of Radiotherapy, Fudan University Shanghai Cancer Center, Shanghai, China

**Keywords:** ALK, non-small-cell lung cancer, crizotinib, progression patterns, sequential therapy beyond crizotinib

## Abstract

**Purpose:** Our study aimed to provide data on effectiveness, safety of crizotinib treatment, brain metastases, progression patterns, and sequential therapy beyond crizotinib treatment in patients with advanced *ALK*-positive NSCLC in China.

**Methods:** We reviewed the medical records of crizotinib-treated NSCLC patients with *ALK*-rearrangement between May 2014 and May 2018 at Fudan University Shanghai Cancer Center. All patients received crizotinib with 250 mg twice daily. Main outcome measures were progression-free survival (PFS), objective response rate (ORR), disease control rate (DCR), the second PFS (PFS2), overall survival (OS), and adverse events.

**Results:** One hundred and four patients with *ALK*-positive NSCLC were included in this retrospective study. ORR and DCR were 82.7 and 98.1%, respectively. The estimated PFS and OS were 13.0 months (95% CI 9.0–17.0 months) and 36.0 months (95% CI 31.0–41.0 months), respectively. Multivariable analysis showed that young age, presence of baseline adrenal gland metastases and non-adenocarcinoma were independent predictive factors for poorer PFS. Presence of baseline adrenal gland metastases, non-adenocarcinoma, intrathoracic progression and shorter crizotinib treatment time were associated with worse OS. Patients without baseline brain metastases (BBM) who were administered with crizotinib as first-line therapy can achieve a significantly longer PFS than those who received crizotinib as second or later line therapy (*p* = 0.006). For patients with BBM receiving sequential therapy beyond the first disease progression after crizotinib treatment (1st PD), crizotinib beyond progressive disease (CBPD) plus local therapy can lead to a significantly longer PFS2 (67.0 vs. 21.0 weeks; *p* = 0.046). Additionally, the OS was significantly longer in patients achieving 1st PD who received CBPD plus local therapy than those who did not receive CBPD or local therapy (35.0 vs. 24.0 months, *p* = 0.041). Presence of brain metastases at any time was in association with worse PFS. No unexpected adverse effects were reported.

**Conclusions:** Crizotinib was effective and well tolerated in Chinese patients with *ALK*-positive, advanced NSCLC in real-world clinical practice. For patients without BBM, crizotinib as first-line therapy can lead to a longer PFS than second-or later line therapy. CBPD plus local therapy after 1st PD beyond crizotinib is feasible and effective in clinical routine practice.

## Introduction

Non-small-cell lung cancer (NSCLC) accounts for 80-85% of all lung cancer (1). Approximately 3–5% of NSCLC patients harbor a rearrangement of the ALK gene, resulting in ~40,000 new cases worldwide per year (2). In 2007, Soda et al. firstly discovered the ALK gene rearrangement with ELM leading to an in-frame fusion protein with oncogenic activity *in vitro* in NSCLC (3). Patients with *ALK-*positive NSCLC have unique clinical and pathologic characteristics including young age, never or light smoking history, adenocarcinoma histology, and the presence of signet-ring cells, etc. (4).

The emergence and development of epidermal growth factor receptor (EGFR)-targeted tyrosine kinase inhibitors (TKI) led to a new chapter of targeted therapies for lung cancer based on different molecular pathology classifications (5). Anaplastic lymphomakinase (ALK) rearrangement was another biomarker discovered in 2007 and the rapid development of effective ALK-TKIs represented another individualized treatment for advanced NSCLC (6). Crizotinib, the multi-targeted mesenchymal-epithelial transition/hepatocyte growth factor receptor *(MET)/ALK/*c-ros oncogene 1 (*ROS1*) inhibitor, was approved initially by the US Food and Drug Administration (FDA) in 2011 for the treatment of advanced ALK-positive NSCLC based on phase I (profile 1001) and phase II (profile 1005) clinical trials that demonstrated significant ORRs of ~60% and improved PFS of 8 months in pretreated *ALK-*positive patients (7, 8). Subsequently, two randomized phase III trials (PROFILE 1014 and 1007) compared crizotinib with standard chemotherapy, leading to full approval for crizotinib as the standard first-line therapy for advanced *ALK*-positive NSCLC in 2015 (9, 10). Crizotinib was also approved by China food and drug administration (CFDA) for *ALK*-positive patients in January 2013.

Although crizotinib can bring a significant benefit in the management of ALK-positive NSCLC, tumors often relapse during the first 2 years (known as acquired resistance). The central nervous system (CNS) is a common relapse lesion. Around 70% of the patients with CNS metastases at baseline had brain progression, while about 20% of the patients without baseline CNS metastases developed new intracranial sites as manifestation of acquired resistance (11). However, based on the low concentrations of crizotinib in the cerebrospinal fluid (12) and poor penetrance of the molecule through the blood-brain barrier (13), failure in the CNS probably represents a pharmacokinetic issue rather than biologic resistance. Next-generation ALK inhibitors such as ceritinib (14, 15), alectinib (16, 17), brigatinib (18, 19) have shown effectiveness both in the second-line therapy after progression on crizotinib and in the first-line therapy for *ALK*-positive NSCLC. However, ceritinib was approved by CFDA for *ALK*-positive NSCLC patients who had progressed on crizotinib or could not tolerant the toxicity of crizotinib on 31st May, 2018; and alectinib was approved by CFDA for *ALK*-positive NSCLC patients on 12th August, 2018. Consequently, in clinical practice in china before 2018, treatment after CBPD in patients with advanced *ALK*-positive NSCLC remains debatable. Our study aimed to provide detailed information on the effectiveness and safety of crizotinib treatment as well as progression patterns, sequential therapy, PFS2 and OS after crizotinib resistance in patients with advanced *ALK*-positive NSCLC in Chinese real-world clinical routine practice.

## Materials and Methods

### Patients

We reviewed the medical records of crizotinib-treated NSCLC patients with *ALK*-rearrangement between May 2014 and May 2018 at Fudan University Shanghai Cancer Center. Patients were included if they had: (1) been histologically or cytologically diagnosed as advanced or metastatic NSCLC with *ALK* rearrangements; (2) been administrated with crizotinib for more than one month; (3) completed tumor response evaluation for crizotinib at least once; (4) complete medical record. Positivity for *ALK* rearrangements was determined using Ventana IHC (immunohistochemistry), FISH (fluorescent *in situ* hybridization), RT-PCR (reverse transcriptase polymerase chain reaction), or NGS (next-generation sequencing) detection methods. We retrospectively collected clinical data and treatment outcomes from the patients' medical records. The clinical stage was assigned according to the 7th edition of the TNM staging system. Data were cut-off at May 30th, 2019. Patients who were treated with crizotinib combined with chemotherapy, lost to follow-up or who did not complete tumor response assessment were excluded from the study.

This study was approved by the institutional review board of Fudan University, Shanghai Cancer Center.

### Treatment

All patients were treated with oral crizotinib at starting dosage of 250 mg twice daily. The dosage could be reduced to 200 mg twice daily, or either interrupted or permanently discontinued due to the occurrence of adverse events (AEs).

### Effectiveness and Safety Evaluation

Effectiveness was assessed by PFS, PFS2, OS, ORR, and DCR. PFS was defined as the time from initiation of crizotinib therapy to the first disease progression beyond crizotinig (1st PD) or death. Patients alive without progression at the time of analysis were censored at their last follow-up. PFS2 was defined as the time from 1st PD to the second disease progression beyond crizotinib or the next-line systemic therapy or death. OS was defined as the time from diagnosis of stage IIIB/IV NSCLC to death due to any cause. Patients alive at the cutoff date were censored. DCR was defined as the percentage of patients with a complete response (CR), partial response (PR), and stable disease (SD). ORR was defined as the percentage of patients with CRs and PRs. The tumor response was initially assessed after 1 month of crizotinib therapy and subsequently every 2 months using the Response Evaluation Criteria In Solid Tumors (RECIST version 1.1). Patients without brain metastasis and without brain symptoms have chest computed tomography (CT) examination, abdominal B ultrasound examination, where there are metastasis lesions plus CT/ magnetic resonance (MR) examination every 2 months; brain MR/CT every 6 months. Patients with brain metastases have chest CT examination, abdominal B ultrasound examination, brain MR/CT and where there are metastasis lesions plus CT/MR examination every 2 months. Patients with brain symptoms have brain MR/CT examination added on the basis of other examinations.

Adverse events (AEs) were assessed every month according to the National Cancer Institute's Common Terminology Criteria for Adverse Events (CTCAE), version 4.0.

### Statistical Analysis

Data were summarized according to frequency and percentage for categorical variables and by medians and ranges for continuous variables. PFS, PFS2 and OS were estimated by the Kaplan-Meier method, along with hazard ratios (HRs). All outcome measures were calculated with 95% confidence intervals (CIs), which were estimated by use of the Cox proportional hazard model. Differences between baseline clinicopathologic characteristics of the groups were assessed using Pearson's χ^2^ or Fisher's exact test.

Exploratory univariate analyses were performed with a log-rank test. Variables with a *p-*value < 0.1 in the univariate analysis were included in a multivariate analysis by use of Cox multivariate models.

The significance level of statistical tests was set at *p* < 0.05. All expressed *p*-values and CIs were two-tailed. AEs were summarized using percentages and frequency counts. All statistical analyses were conducted using IBM®SPSS® Statistics version 24.

## Results

### Patient Baseline Characteristics

One hundred and forty-eight consecutive advanced or metastatic NSCLC patients with *ALK*-rearrangement were treated at Fudan University Shanghai Cancer Center between May 2014 and May 2018. Twenty-one patients who did not receive crizotinib, 4 patients who were treated with crizotinib combined with chemotherapy, 11 who were lost to follow-up and 8 who did not take crizotinib for at least 1 month, nor complete the tumor response assessment were excluded from the study. A total of 104 patients were eligible for our study, among whom 40 (38.5%) patients presented with baseline brain metastasis (BBM) at the initiation of crizotinib treatment and 64 (61.5%) patients did not have baseline brain metastasis (Non-BBM). Their baseline characteristics at the initiation of crizotinib therapy are summarized in Table 1. The patients' median age was 49.5 years (range, 21 to 84 years), and 87.5% (91/104) were younger than 65 years old. 58.7% (61/104) patients were male and 75.0% (78/104) were never-smokers. The majority of patients (101/104, 97.1%) were diagnosed as adenocarcinoma; 84.6% (88/104) patients were stage IV disease at baseline and 17.3% (18/104) patients were postoperative recurrent disease. All 104 patients had an Eastern Cooperative Oncology Group performance status (ECOG PS) of 0 to 2 at baseline, with 96.2% (100/104) having ECOG PS of 0-1 at baseline.

**Table 1:** Baseline patient characteristics (*n* = 104).

**Characteristic**	**All, No. of patients (%)^a^**	**BBM** **(*n* = 40)**	**Non-BBM (*n* = 64)**	***P*-value**
Age, years				
Mean	49.63	46.48	51.61	0.039
Median	49.50	47.50	51.50	
Range	21–84	21–65	26-84	
Age group				0.077
<65 years	91 (87.5)	38 (95.0)	53 (82.8)	
≥65 years	13 (12.5)	2 (5.0)	11 (17.2)	
Sex				0.001
Male	61 (58.7)	15 (37.5)	46 (71.9)	
Female	43 (41.3)	25 (62.5)	18 (28.1)	
Smoking history				0.486
Never-smoker	78 (75.0)	32 (80.0)	46 (71.9)	
Former or current smoker	26 (25.0)	8 (20.0)	18 (28.1)	
Histology				0.054
Adenocarcinoma	101 (97.1)	37 (92.5)	64 (100.0)	
Neuroendocrine carcinoma	3 (2.9)	3 (7.5)	0	
ECOG PS at baseline				0.056
0	2 (1.9)	2 (5.0)	0	
1	98 (94.2)	35 (87.5)	63 (98.4)	
2	4 (3.8)	3 (7.5)	1 (1.6)	
Stage at baseline				0.000
IIIB	16 (15.4)	0	16 (25.0)	
IV	88 (84.6)	40 (100.0)	48 (75.0)	
Postoperative recurrent	18 (17.3)	6 (15.0)	12 (18.6)	
Metastatic sites at baseline				
Lung	18 (17.3)	9 (22.5)	9 (14.1)	0.296
Brain	40 (38.5)	40 (100.0)	0	
Bone	42 (40.4)	16 (40.0)	26 (40.6)	1.000
Liver	13 (12.5)	7 (17.5)	6 (9.4)	0.239
Adrenal gland	11 (10.6)	4 (10.0)	7 (10.9)	1.000
Supraclavicular lymph node	39 (37.5)	9 (22.5)	30 (46.9)	0.014
Pleural	34 (32.7)	9 (22.5)	25 (39.1)	0.090
Others	20 (19.2)	5 (12.5)	15 (23.4)	0.207
No. of metastatic sites				0.008
0	16 (15.4)	0	16 (25.0)	
1	36 (34.6)	13 (32.5)	23 (35.9)	
2	27 (26.0)	13 (32.5)	14 (21.9)	
≥3	25 (24.0)	14 (35.0)	11 (17.2)	
*ALK* detection methods				0.482
IHC and FISH	34 (32.7)	16 (40.0)	18 (28.1)	
FISH only	26 (25.0)	11(27.5)	15 (23.4)	
IHC only	34 (32.7)	10 (25.0)	24 (37.5)	
RT-PCR	4 (3.8)	2 (5.0)	2 (3.1)	
NGS	6 (5.8)	1 (2.5)	5 (7.8)	
Lines of crizotinib therapy				0.647
1	63 (60.6)	24 (60.0)	39 (60.9)	1.000
2	30 (28.8)	13 (32.5)	17 (26.6)	
≥3	11 (10.6)	3 (7.5)	8 (12.5)	

a *Unless otherwise stated*.

*ECOG PS, Eastern Cooperative Oncology Group performance status; FISH, fluorescent in situ hybridization; NGS, next-generation sequencing; RT-PCR, reverse transcriptase polymerase chain reaction; BBM, baseline brain-metastases; Non-BMM, without baseline brain-metastases*.

Most patients (88/104, 84.6%) had a distant metastasis at baseline. The most common metastatic sites were bone (40.4%), brain (38.5%), supraclavicular lymph node (37.5%) and pleural (32.7%). In 63 (60.6%) patients, crizotinib was used as first-line treatment, 30 (28.8%) as second-line treatment, and 11 (10.6%) as third-line or later treatment. 90.4% (94/104), 3.8% (4/104), and 5.8% (6/104) patients were determined as *ALK* rearrangements using Ventana IHC and/or FISH, RT-PCR, or NGS detection, respectively.

The baseline characteristics of patients were compared between the BBM and non-BBM groups (Table 1). There were more female patients in BBM group than those in the non-BBM group. Other features, including age, smoking history, ECOG PS score, and histology were not significantly different between the two groups (Table 1). In addition, multivariate analyses of logistic regression revealed that women were more likely to have BBM (HR = 0.210; 95% CI, 0.074–0.597; *p* = 0.003) (Table 2). Among 40 patients with BBM, 6 patients received surgical therapy for brain metastases and 10 patients underwent the whole brain radiotherapy (WBRT) before crizotinib treatment. The other 24 patients did not receive any local therapy for brain metastases before crizotinib treatment.

**Table 2:** Cox regression analysis of factors associated with baseline brain metastases (*n* = 104).

**Variable**	**Univariable analysis *P-*value**	**Multivariable analysis**	
		**Hazard ratio** **[95% CI]**	***P-*value**
Sex (female vs. male)	0.001	0.210 [0.074–0.597]	0.003
Age (≥65 vs. <65 years)	0.077	0.274 [0.049–1.516]	0.138
Smoking history (yes vs. no)	0.486	1.578 [0.488–5.101]	0.446
ECOG PS at baseline (<2 vs. ≥2)	0.157	0.415 [0.029–5.924]	0.516
Adenocarcinoma (yes vs. no)	0.054	NA	0.999

### Tumor Responses

Tumor responses are shown in Table 3. Eighty-six patients (82.7%) achieved PR and 16 patients (15.4%) had SD, resulting in an ORR of 82.7% (95% CI, 75.0–90.0%) and a DCR of 98.1% (95% CI, 95.0–101.0%). Two patients (5.7%) who reported PD as the best response had disease progression after 1 month of crizotinib treatment. Among 40 patients with BBM, the ORR was 85.0%, with 34 patients achieving PR, and the DCR was 100%, with 6 patients having SD. In 64 patients without BBM, the ORR was 81.3%, with 52 patients achieving PR, and the DCR was 96.9%, with 10 patients having SD. There were no statistically significant differences of ORR and DCR between the two groups (*p* = 0.791; *p* = 0.522).

**Table 3:** Tumor responses.

**Responses**	**All patients** **(*n* = 104) [n or %]**	**BBM** **(*n* = 40) [n or %]**	**Non-BBM** **(*n* = 64) [n or %]**
CR	0 (0.0)	0	0
PR	86 (82.7)	34 (85.0)	52 (81.3)
SD	16 (15.4)	6 (15.0)	10 (15.6)
PD	2 (1.9)	0	2 (3.1)
ORR	82.7% [95% CI, 75–90%]	85.0%^*^	81.3%
DCR	98.1% [95% CI, 95–101%]	100%^**^	96.9%

* *P = 0.791 vs. Non-BBM*.

** *P = 0.522 vs. Non-BBM*.

*CR, complete response; DCR, disease control rate; ORR, objective response rate; PD, progressive disease; PR, partial response; SD stable disease; BBM, baseline brain-metastases; Non-BMM, without baseline brain-metastases*.

### Analysis of Progression-Free Survival Time

**Figure 1:**
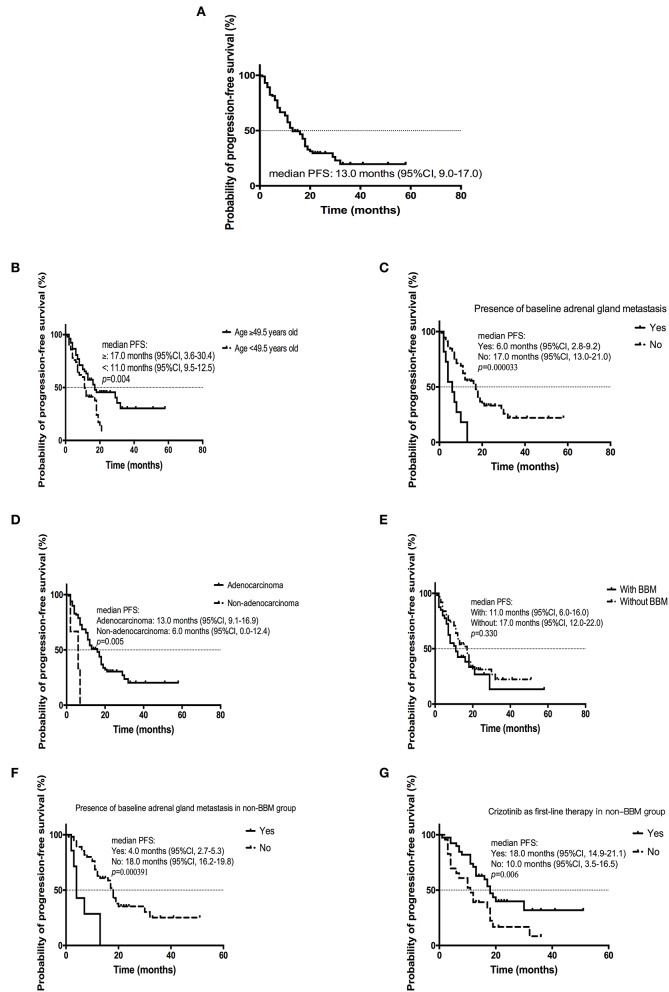
Kaplan-Meier curve of PFS **(A)** of all patients (*n* = 104); **(B)** of patients age ≥ 49.5 years old or <49.5 years old; **(C)** of patients with or without baseline adrenal gland metastasis; **(D)** of patients diagnosed with adenocarcinoma or non-adenocarcinoma; **(E)** of patients with or without BBM; **(F)** of patients with or without baseline adrenal gland metastasis in non-BBM group; **(G)** of patients administered with crizotinib as first-line therapy or second or later line in non-BBM group.

By the cutoff day (May 30th, 2019), 70 (67.3%) patients developed disease progression. The estimated median PFS was 13.0 months (95% CI, 9.0 to 17.0 months; Figure 1A). Several factors were analyzed to predict the PFS with crizotinib. A log-rank test demonstrated that age (*p* = 0.004), treatment line (*p* = 0.036), metastatic sites (*p* = 0.003), presence of baseline adrenal gland metastases (*p* = 0.000033), and histology (*p* = 0.005) were significantly associated with PFS. On multivariable analysis, younger patients (age ≥49.5 vs. <49.5 years, HR = 0.415, 95% CI [0.242–0.713]; *p* = 0.001), presence of baseline adrenal gland metastases (yes vs. no, HR = 3.922, 95% CI [1.803–8.529]; *p* = 0.001) and non-adenocarcinoma (adenocarcinoma vs. others, HR = 0.242, 95% CI [0.068–0.862]; *p* = 0.029) were independent risk factors for poorer PFS (Table 4; Figures 1B–D).

**Table 4:** Cox multivariate analysis of progression-free survival in all patients (*n* = 104).

**Variables**	**Log-rank** **tesk**	**Multivariate analysis**		
		**HR**	**95% CI**	***P***
Sex (male vs. female)	0.142			
Age (≥49.5 years vs. <49.5 years)	0.004	0.415	0.242–0.713	0.001
Smoking history (yes vs. no)	0.140			
Brain metastasis (yes vs. no)	0.330			
Bone metastasis (yes vs. no)	0.175			
Lung metastasis (yes vs. no)	0.101			
Liver metastasis (yes vs. no)	0.109			
Adrenal gland metastasis (yes vs. no)	0.000033	3.922	1.803–8.529	0.001
Supraclavicular lymph node metastasis (yes vs. no)	0.931			
Pleural metastasis (yes vs. no)	0.475			
Metastasis (yes vs. no)	0.193			
Metastasis (≥2 sites vs. <2 sites)	0.003	1.460	0.859–2.480	0.162
Adenocarcinoma (yes vs. no)	0.005	0.242	0.068–0.862	0.029
Crizotinib as first-line therapy (yes vs. no)	0.036	0.734	0.444–1.211	0.226

The estimated mPFS were 11.0 months (95% CI, 6.0–16.0 months) and 17.0 (95% CI, 12.0–22.0 months) for patients with or without BBM, respectively; although the difference was not statistically significant (*p* = 0.330) (Figure 1E). For patients without BBM, absence of baseline adrenal gland metastases (no vs. yes, HR = 5.323, 95% CI [1.876–15.104]; *p* = 0.002) and crizotinib treatment line (first vs. second-or later, HR = 0.474, 95% CI [0.226–0.993]; *p* = 0.048) were independent predictors of longer PFS after multivariable analysis (Figures 1F,G). For patients with BBM, there was no independent factor for PFS after multivariate analysis.

### Progression Patterns and Sequential Therapy Beyond Crizotinib Resistance

The progression patterns of 70 patients who experienced the first disease progression beyond crizotinib (1st PD) are shown in Table 5. Twenty-three patients (23/104, 22.1%) developed PD in new lesions, 34 patients (34/104, 32.7%) developed regrowth of previous lesions and 13 (13/104, 12.5%) patients developed progressive disease in more than one site. Thirty-four (32.7%) patients developed progressive disease in brain, 23 (22.1%) patients developed intrathoracic disease progression and 14 (13.5%) patients developed PD in bone. There were no significant differences both in number of patients experiencing PD (*p* = 1.000) and in progressive patterns (*p* = 0.099) between patients with or without BBM. There was a numerically higher intracranial progression rate in patients with BBM (42.5%) than that in patients without BBM (26.6%) (*p* = 0.085). By the time of analysis, 57 (54.8%) patients had brain metastases.

**Table 5:** Progression patterns.

		**All** **(*N* = 1)** **n (%)**	**BBM** **(*N* = 40)** **n (%)**	**Non-BBM** **(*N* = 6)** **n (%)**
Patients develop progressive disease during crizotinib therapy		70 (67.3)	27 (67.5)	43 (67.2)
Progressive patterns	1#	23 (22.1)	5 (12.5)	18 (28.1)
	2#	34 (32.7)	17 (42.5)	17 (26.6)
	3#	13 (12.5)	5 (12.5)	8 (12.5)
Intracranial progression		34 (32.7)	17 (42.5)^*^	17 (26.6)

* *P = 0.085 vs. Non-BBM*.

*1#, New lesions; 2#, Regrowth of previous lesions; 3#, More than one site; BBM, baseline brain-metastases; Non-BMM, without baseline brain-metastases*.

**Figure 2:**
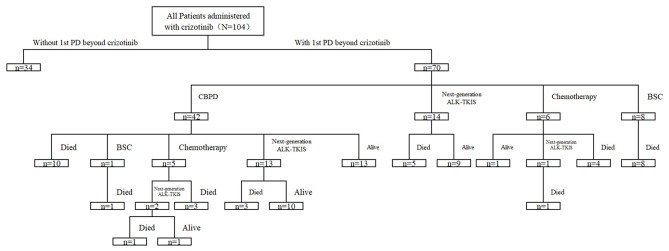
Sequential therapy after the first disease progression (1st PD) beyond crizotinib.

Among all patients experiencing 1st PD, 42 (42/70, 60.0%) patients continued crizotinib beyond 1st PD (CBPD) for > 3 weeks (median 11 weeks), 14 (14/70, 20%) switched to next-generation ALK-TKIs, 6 (6/70, 8.6%) received chemotherapy, and the other 8 (8/70, 11.4%) received best supportive care (BSC) until the second disease progression beyond crizotinib (2nd PD) (Figure 2). Sequential therapy is shown in Figure 2. Twenty-nine patients received local therapy after disease progression; 24 received radiotherapy for brain disease (17 received WBRT, 4 received stereotactic body radiotherapy, 3 received surgery), three received radiotherapy for thoracic progression, two received palliative radiotherapy for bone metastases, one received surgical therapy for bone metastases and one received radiofrequency ablation for liver metastases.

By the time of analysis, 47 (45.2%) patients were still administered with crizotinib and the median duration of crizotinib treatment was 16.0 months.

### PFS2 and OS in Patients Receiving 1st PD Beyond Crizotinib

**Figure 3:**
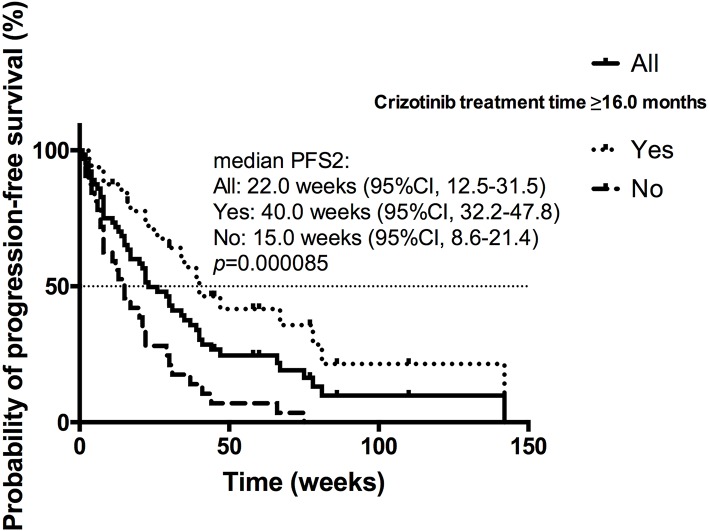
Kaplan-Meier curve of the PFS2 of all patients receiving 1st PD beyond crizotinib (*n* = 70), of patients received crizotinib treatment ≥16.0 or <16.0 months.

Among the patients receiving 1st PD, the estimated median PFS2 (mPFS2) was 22.0 weeks (95% CI 12.5–31.5) for all 70 patients (Figure 3). The univariate analysis found that presence of brain metastases at present (*p* = 0.033), local therapy for brain metastases after crizotinib (*p* = 0.098), intracranial progression (*p* = 0.036), duration of crizotinib treatment (*p* = 0.000085) and CBPD plus local therapy (*p* = 0.091) were in association with PFS2. On multivariable analysis, longer crizotinib treatment (≥16.0 vs. <16.0 months; 15.0 vs. 40.0 weeks; HR = 0.317; 95% CI 0.150–0.668; *p* = 0.003) was the only independently predictive factor for longer PFS2 (Figure 3). Among these 70 patients, 27 patients had baseline brain metastases. Among these 27 patients with BBM, 19 patients received CBPD, 13 patients received local therapy after 1st PD (WBRT for 9 patients, stereotactic body radiotherapy for 2 patients, surgery for 2 patients), and total 11 patients attained CBPD plus local therapy after 1st PD. For patients with BBM, PFS2 was significantly longer in 11 patients who attained CBPD plus local therapy after progression (67.0 vs. 21.0 weeks; *p* = 0.046) than the other 16 patients who did not receive CBPD or local therapy.

The estimated median OS (mOS) was 29.0 months (95% CI 22.0–36.0) for all 70 patients achieving 1st PD beyond crizotinib. Among these 70 patients, 42 patients received CBPD, 29 received local therapy after 1st PD (details are above), and total 24 patients received CBPD plus local therapy. Compared with the other 46 patients receiving 1st PD beyond crizotinib who did not receive CBPD or local therapy, 24 patients receiving 1st PD beyond crizotinib can still obtain a significant OS benefit from CBPD plus local therapy (35.0 vs. 24.0 months, *p* = 0.041).

### Analysis of Overall Survival Time of All Patients

**Figure 4:**
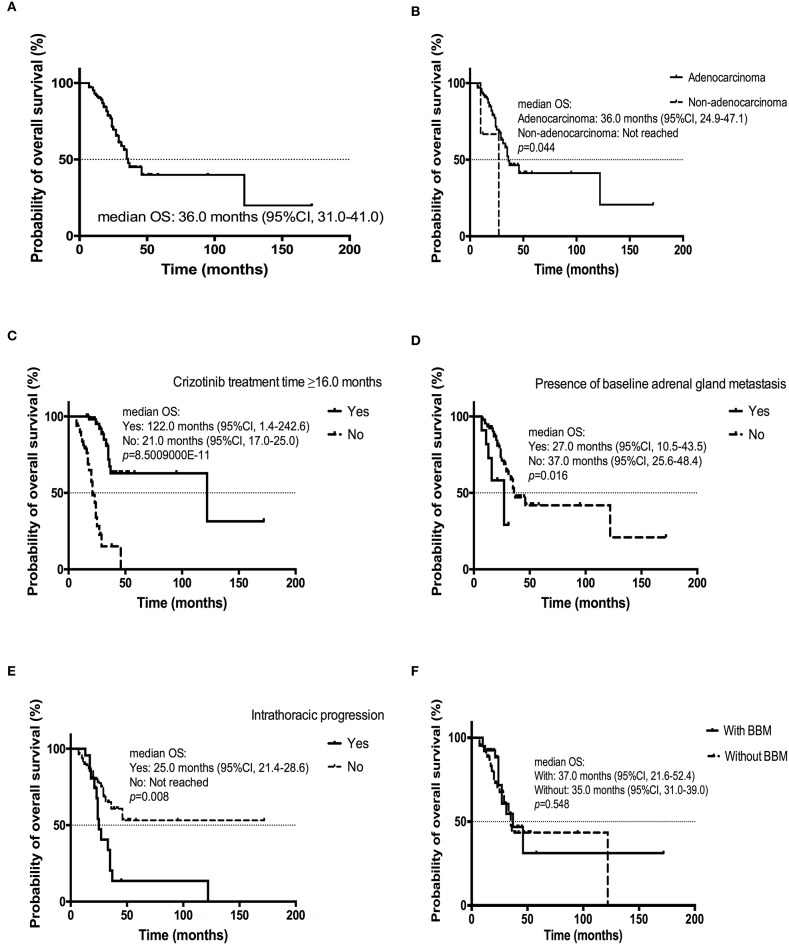
Kaplan-Meier curve of OS **(A)** of all patients (*n* = 104); **(B)** of patients diagnosed with adenocarcinoma or non-adenocarcinoma; **(C)** of patients received crizotinib treatment ≥16.0 or < 16.0 months; **(D)** of patients with or without baseline adrenal gland metastasis; **(E)** of patients experiencing intrathoracic progression or not; **(F)** of patients with or without BBM.

The estimated median OS was 36.0 months (95% CI, 31.0–41.0 months; Figure 4A). Multivariate Cox analysis revealed that adenocarcinoma (yes vs. no, HR = 0.159, 95% CI [0.031–0.798]; *p* = 0.026) and longer crizotinib treatment (≥16.0 vs. <16.0 months, HR = 0.123, 95% CI [0.050–0.305]; *p* = 8.5009000E-11) were favorable predictors for improved OS (Table 6; Figures 4B,C). Additionally, presence of baseline adrenal gland metastases (yes vs. no, HR = 3.410, 95% CI [1.068–10.887]; *p* = 0.035) and intrathoracic progression (yes vs. no, HR = 3.112, 95% CI [1.374–7.049]; *p* = 0.007) were independent risk factors for poorer OS (Table 6; Figures 4D,E).

**Table 6:** Cox multivariate analysis of overall survival in all patients (*n* = 104).

**Variables**	**Log-rank** **tesk**	**Multivariate analysis**		
		**HR**	**95% CI**	***P***
Sex (male vs. female)	0.554			
Age (≥49.5 years vs. <49.5 years)	0.077	0.646	0.306–1.361	0.250
Smoking history (yes vs. no)	0.118			
Brain metastasis (yes vs. no)	0.548			
Bone metastasis (yes vs. no)	0.375			
Lung metastasis (yes vs. no)	0.817			
Liver metastasis (yes vs. no)	0.022	1.637	0.473–5.663	0.436
Adrenal gland metastasis (yes vs. no)	0.016	3.431	1.088–10.822	0.035
Supraclavicular lymph node metastasis (yes vs. no)	0.967			
Pleural metastasis (yes vs. no)	0.173			
Metastasis (yes vs. no)	0.314			
Metastasis (≥4 sites vs. <4 sites)	0.037	0.637	0.151–2.681	0.539
Adenocarcinoma (yes vs. no)	0.044	0.159	0.031–0.798	0.026
Crizotinib as first-line therapy (yes vs. no)	0.406			
Tumor responses (CR/PR vs. SD/PD)	0.0002990	0.552	0.251–1.215	0.140
Intracranial progression (yes vs. no)	0.909			
Intrathoracic progression (yes vs. no)	0.008	3.112	1.374–7.049	0.007
Bone progression (yes vs. no)	0.0001670	2.604	0.993–6.827	0.052
Crizotinib treatment time (≥16 vs. <16 months)	8.5009000E−11	0.123	0.050–0.305	0.0000060

There was no significant difference in mOS for patients with or without BBM (37.0 months, 95% CI, 21.6–52.4 months; 35.0 months, 95% CI, 31.0–39.0 months; *p* = 0.548) (Figure 4F). Longer crizotinib treatment (≥16.0 vs. <16.0 months) was also significantly associated with a longer OS in patients with or without BBM (*p* = 0.005; *p* = 0.000072).

### Safety

Elevated transaminases (48/104, 46.2%), elevated blood creatinine (28/104, 26.9%), neutropenia (20/104, 19.2%), diarrhea (20/104, 19.2%) were the most commonly reported crizotinib-related adverse effects (AEs) (Table 7). Eight patients reported crizotinib-related grade 3-5 AEs (7.7%), including 4 patients with grade 3 elevated transaminases, 1 grade 4 elevated transaminases and 3 grade 3 neutropenia. No unexpected AEs were observed. One patient discontinued crizotinib because of grade 4 elevated transaminases. Two patients were once administered to 200 mg twice daily due to grade 3 elevated transaminases.

**Table 7:** Adverse events reported (*n* = 104).

**Adverse events**	**All grades** **No. (%)**	**≥Grade 3** **No. (%)**
Any	80 (76.9)	8 (7.7)
Elevated transaminases	48 (36.2)	5 (4.8)
Elevated blood creatinine	28 (26.9)	
Diarrhea	20 (19.2)	
Neutropenia	20 (19.2)	3 (2.9)
Edema	18 (17.3)	
Leukopenia	18 (17.3)	
Vision disorder	13 (12.5)	
Anemia	13 (12.5)	1 (1.0)
Vomiting	11 (10.6)	
Constipation	10 (9.6)	
Decreased appetite	9 (8.7)	
Fatigue	7 (6.7)	
Dysgeusia	5 (4.8)	
Nausea	5 (4.8)	
Rash	3 (2.9)	
Thrombocytopenia	1 (1.0)	

## Discussion

Our retrospective study provided first-hand data of the effectiveness and safety of crizotinib treatment, brain metastases, progression patterns and sequential therapy beyond crizotinib in patients with advanced *ALK*-positive NSCLC in Chinese routine clinical practice at Fudan University Shanghai Cancer Center.

*ALK*-rearrangement has been found to be associated with several distinctive clinicopathologic features including slightly more male, younger age, adenocarcinoma histology, absence of smoking history and absence of other oncogenic drivers (4, 20). The demographic characteristics of patients in our study were consistent with the findings before. We found that women might be more likely to develop brain metastases than men (58.1 vs. 24.6%, *p* = 0.003). There are few studies on which female patients with *ALK*-positive NSCLC are more likely to develop brain metastases. It requires a larger sample size to verify our result.

In phase I and phase II studies, crizotinib achieved ORR of 60% and mPFS of 7–10 months in heavily pretreated *ALK*-positive NSCLC patients (7, 8, 21). Crizotinib also provided a significant prolonged PFS (10.9 vs. 7.4 months, *P* < 0.0001), and higher ORR (74 vs. 45%, *P* < 0.001) compared with platinum-based doublet chemotherapy in the first line setting in the phase III study PROFILE 1014. The ORR (82.7%), DCR (98.1%) and mPFS (13.0 months) in our study were slightly higher than the results of clinical trials. It can be explained that our study had a higher proportion of IIIB (16/104, 15.4%) and ECOG PS of 0-1 (100/104, 96.2%) than these clinical trials. The OS was shorter in our study than that in other studies (22, 23). Several factors may account for it. Firstly, because of unavailability of next-generation ALK-TKIs in China at that time, few patients can receive next-generation ALK-TKIs after crizotinib failure. Secondly, a number of patients who received first-line crizotinib may have missed the opportunity to receive chemotherapy because they continued taking ALK-TKIs for too long after disease progression or they just refused to receive chemotherapy and the efficacy of chemotherapy was not satisfactory. Thirdly, after all, the OS data in our study was not mature.

There were some studies exploring independent predictors of PFS and OS in ALK-positive NSCLC patients during *ALK*-TKI treatment. Pailler et al. (24) found that “smoking status (≥15 vs. <15 pack-year), number of previous treatment (≥2 vs. <1), number of metastatic sites (≥2 vs. <1), and dynamic change of *ALK*-copy number gain (CNG) circulating tumor cell counts (stable/increase vs. decrease) were independent predictive factors for PFS”. Johung et al. (25) found that “absence of extracranial metastases, Karnofsky performance score ≥90, and no history of TKIs before development of brain metastases were associated with improved survival in ALK positive patients with brain metastasis.” In Xu's study (26), “long PFS with crizotinib (≥10.4 months), intracranial progression, and use of next-generation ALK inhibitors might be favorable predictors for OS in advanced ALK-positive NSCLC patients.”

Multivariable analysis of our study showed that young age, presence of baseline adrenal gland metastases and non-adenocarcinoma were independent predictive factors for poorer PFS. In addition, presence of baseline adrenal gland metastases, non-adenocarcinoma, intrathoracic progression and shorter crizotinib treatment time were prognostic factors for worse OS. It is worth noting that since there were only three cases of non-adenocarcinoma, this result requires a larger sample to confirm. It cannot be ignored that crizotinib treatment time may be a trick factor for OS. There were no common predictive factors for PFS and OS between our research and previous studies, though the result that BBM did not influence PFS or OS was consistent with the findings of Pacheco et al. (26). Interestingly, for patients without BBM, crizotinib treatment line was an independent predictor of longer PFS after multivariable analysis. Patients without BBM who were administered with crizotinib as first-line therapy can achieve a significantly longer PFS than those as second or later line therapy. Although next-generation ALK-TKIs such as alectinib, brigatinib demonstrated superior PFS vs. crizotinib in untreated *ALK*-positive NSCLC regardless of baseline brain metastases (26–28), the difference in OS of patients who received crizotinib or next-generation ALK-TKIs as first-line therapy is still unknown.

Although a significant benefit can be achieved in the management of *ALK*-positive NSCLC with crizotinib, it is worth noting that a substantial risk of central nervous system (CNS) progression inevitably exists (13, 29). The patients with *ALK*-positive NSCLC have a high risk of developing CNS metastasis, as observed in ~30% of cases at the time of tumor diagnosis and in 50–60% of patients during crizotinib treatment (30). Our study analyzed brain metastases and progression pattern and found that brain metastases occurred in 32.7% of patients (34 of 104) with PD at the time of data cutoff during crizotinib therapy. The rate of intracranial progression was numerically higher (*p* = 0.085) in patients with BBM (17/40, 42.5%) than that in patients without BBM (17/64, 26.6%) during the period of crizotinib therapy. In any case, *ALK*-positive patients are prone to brain metastases, which may be related to the low penetration to CNS of crizotinib, the strong driving force of *ALK* gene, the addiction to CNS of *ALK*-positive tumors and the prolongation of survival after the use of crizotinib.

So far, few studies focused on progression patterns, sequential therapy and survival in patients achieving 1st PD beyond crizotinib. Our study clarified these issues in detail. For patients with BBM achieving 1st PD, CBPD plus local therapy can lead to a significantly longer PFS2 (67.0 vs. 21.0 weeks; *p* = 0.046). Additionally, CBPD plus local therapy can significantly extend OS in patients achieving 1st PD beyond crizotinib (35.0 vs. 24.0 months, *p* = 0.041). In Pacheco et al.'s study (26), they defined patients with all progressing lesions treated with local ablative therapy (LAT) while continuing to take crizotinib as having oligoprogressive disease (OPD) treated with LAT. They found that LAT for OPD was not associated with improved OS, although there was a trend toward benefit (HR = 0.58, *p* = 0.14). Local therapy in our study covered not only local ablative therapy, but also local surgical therapy and radiotherapy. Thus, CBPD plus local therapy after 1st PD beyond crizotinib is feasible and effective in clinical practice.

There were several limitations of our study. As a retrospective study, information bias could have had an impact on the outcomes of our study. On the other hand, selection bias caused by loss of follow-up, single-center studies, and limitations of sample representation were inevitable. Lastly, due to unavailability of next-generation ALK TKIs such as ceritinib and alectinib before May 2018 in China, we only reviewed the medical records of crizotinib-treated NSCLC patients with *ALK*-rearrangement between May 2014 and May 2018 in our institute. Further research on next-generation ALK inhibitors are needed since they have been proven superior both in the second-line therapy after progression on crizotinib and in the first-line therapy for *ALK*-positive NSCLC.

## Conclusions

In conclusion, our study found that crizotinib was effective and well tolerated in Chinese patients with *ALK*-positive, advanced NSCLC in real-world routine clinical practice. Young age, presence of baseline adrenal gland metastases and non-adenocarcinoma were independent predictive factors for poorer PFS. Presence of baseline adrenal gland metastases, non-adenocarcinoma, intrathoracic progression and shorter crizotinib treatment time were associated with worse OS. For patients without BBM, crizotinib as first-line therapy can lead to a longer PFS than second or later line. CBPD plus local therapy after 1st PD beyond crizotinib is feasible and effective in clinical routine practice.

## Data Availability Statement

All datasets analyzed for this study are included in the manuscript and the supplementary files.

## Ethics Statement

The studies involving human participants were reviewed and approved by Fudan University Shanghai Cancer Center. The patients/participants provided their written informed consent to participate in this study.

## Author Contributions

JW and JC designed the study, collected data and verified its integrity, and helped write the manuscript. CL and HY were responsible for statistical analysis and writing the manuscript. QL, HC, YL, WZ, KZ, ZZ, SS, and MF were responsible for collecting and verifying the integrity of the data. All authors critically reviewed the manuscript, and all approved the final version submitted for publication.

### Conflict of Interest

The authors declare that the research was conducted in the absence of any commercial or financial relationships that could be construed as a potential conflict of interest.

## Abbreviations

AE: adverse events
ALK: anaplastic lymphoma kinase
CBPD: crizotinib beyond progressive disease
CI: confidence interval
CR: complete response
DCR: disease control rate
ECOG PS: Eastern Cooperative Oncology Group performance status
EGFR: epidermal growth factor receptor
FISH: fluorescent *in situ* hybridization
HR: hazard ratio
MET: mesenchymal-epithelial transition/hepatocyte growth factor receptor
NSCLC: non–small-cell lung cancer
NGS: next-generation sequencing
ORR: objective response rate
OS: overall survival
PD: progressive disease
PFS: progression-free survival
PR: partial response
RECIST: response evaluation criteria in solid tumors
CT: computed tomography
MR: magnetic resonance
*ROS1*: c-ros oncogene 1
RT-PCR: reverse transcriptase polymerase chain reaction
SD: stable disease
TKI: tyrosine kinase inhibitor
TNM: tumor-node-metastasis
WBRT: whole-brain radiotherapy
BBM: with baseline brain-metastases
Non-BBM: without baseline brain-metastases.
